# Mature B‐cell lymphomas in adolescents and young adults

**DOI:** 10.1002/jha2.783

**Published:** 2023-09-08

**Authors:** Nader Kim El‐Mallawany, Lisa Giulino‐Roth, John M. Burke, Michelle Hermiston, Carl E. Allen

**Affiliations:** ^1^ Department of Pediatrics, Baylor College of Medicine, Texas Children's Hospital Texas Children's Cancer Center Houston Texas USA; ^2^ Department of Pediatrics Weill Cornell Medical College New York New York USA; ^3^ Department of Hematology Rocky Mountain Cancer Centers Aurora Colorado USA; ^4^ Department of Pediatrics University of California San Francisco California USA

**Keywords:** adolescent, DLBCL, mature B cell lymphoma, PMBCL, young adult

## Abstract

Pediatric non‐Hodgkin lymphoma includes over 30 histologies (many with subtypes), with approximately 800 cases per year in the US, compared to >60,000 cases of adult NHL annually. Improvements in survival in pediatric and adolescent mature B cell NHL over the past 5 decades align with the overall success of the cooperative trial model with dramatic improvements in outcomes through dose escalation of chemotherapy and, more recently, targeted therapy with rituximab. Pediatric dose‐intense strategies carry risks of long‐term consequences, but treatment failure is nearly universally fatal. By comparison, adult mature B cell lymphoma is typically less aggressive and treated with less intense chemotherapy. Optimizing therapy for adolescents and young adults remains a major challenge that requires creative solutions, including engineering study groups to combine biologically comparable adult and pediatric populations and developing effective salvage strategies that will ultimately be required for investigations of front‐line dose reduction. In this review, we discuss challenges and opportunities for improving outcomes for adolescents and young adults with high‐grade mature B cell lymphomas, diffuse large B cell lymphoma, and primary mediastinal B cell lymphoma.

## 1 HIGH‐GRADE MATURE B CELL LYMPHOMA IN AYA

Non‐Hodgkin lymphoma (NHL) refers to a broad and heterogenous group of lymphomas that share the feature of being distinct from Hodgkin lymphoma [1]. There are approximately 800 cases of pediatric NHL in the United States annually, compared to over 60,000 cases of adult NHL [2]. Compared to adult mature B cell lymphoma, the majority in children are high‐grade, arising from immune accidents in germinal centers, with increased risk in children and adolescents with immune disorders [3]. This review will focus on mature B‐cell lymphomas occurring in adolescents and young adults (AYA), encompassing the age range from 15–39 years. This AYA subset of the population is characterized by shared epidemiological and clinical patterns that are distinct from younger children and older adults and potentially linked by shared disease biology. The landscape of mature high‐grade B‐cell lymphomas (HGBL) in AYA is primarily composed of Burkitt lymphoma in childhood and early adolescence, with diffuse large B‐cell lymphoma (DLBCL) increasing in incidence through adolescence and young adulthood [4]. The gray‐zone histologies filling in the spectrum between Burkitt and DLBCL have evolved with each iteration of the WHO classification of lymphoid neoplasms [5]. (Table 1) Previously conceptualized as Burkitt‐like lymphomas, lack of *C‐MYC* rearrangement or characteristic histological features distinguishes them. The 2022 version of the WHO classification names these entities HGBL with 11q aberrations, HGBL with *MYC* and *BCL2* rearrangements, and HGBL, NOS [6]. The precise association and molecular epidemiology of these gray‐zone entities in the pediatric and AYA population though, remains uncertain. Meanwhile, there has been expansive growth in defining seven genetic subtypes of DLBCL in adults, illuminating distinctions in the translational biology forming the basis of the phenotypic and clinical heterogeneity that characterizes adult DLBCL [7]. Landmark discovery in the realm of lymphoma genomic biology has yet to occur in pediatric/AYA DLBCL leaving the landscape largely unexplored and incompletely understood [8]. As such, the spectrum of pediatric lymphomas ranging from Burkitt to gray‐zone HGBL to DLBCL is treated uniformly regardless of underlying histology [4].

**TABLE 1:** Categorization of B‐cell non‐Hodgkin lymphomas and lymphoproliferative disorders in the context of cell origin and incidence specific to the pediatric/adolescent population.

Category	Histology
Common	Burkitt lymphoma
	Diffuse large B‐cell lymphoma (DLBCL), NOS
	Primary mediastinal B‐cell lymphoma
Less common and rare	B‐cell lymphoblastic lymphoma
	B‐cell PTLD (non‐destructive, polymorphic, monomorphic)
	High‐grade B‐cell lymphoma, NOS
	High‐grade B‐cell lymphoma with 11q aberrations
	High‐grade B‐cell lymphoma with *MYC* and *BCL2* rearrangements
	Large B‐cell lymphoma with *IRF4* rearrangement
	T‐cell/histiocyte‐rich Large B‐cell lymphoma
	Mediastinal gray‐zone lymphoma ^*^
	Pediatric‐type follicular lymphoma
	Pediatric nodal marginal zone lymphoma
Very rare	Extranodal marginal zone lymphoma
	EBV+ DLBCL, NOS
	Lymphomatoid granulomatosis
	Primary CNS lymphoma
	Plasmablastic lymphoma
	Primary effusion lymphoma
	Multicentric Castleman disease

Abbreviations: CNS, central nervous system, EBV, Epstein‐Barr virus, NOS, not otherwise specified; PTLD, posttransplant lymphoproliferative disorders

* Also previously known as: B‐cell lymphoma, unclassifiable, with features intermediate between DLBCL and classical Hodgkin lymphoma.

### 1.1 Treatment of HGBL in AYA

In the era of rituximab, curative outcomes for HGBL on pediatric regimens approach 95% [9]. Generally, the two widely utilized chemotherapy regimens in high‐income countries were devised by the Lymphomes Malins B (LMB) and Berlin‐Frankfurt‐Münster (BFM) consortia [4]. These treatment regimens offer a risk‐stratified approach that categorizes patients into low (LMB Group A, BFM R1), intermediate (Group B/R2 and R3), and high‐risk (Group C/R4) strata based on disease stage and lactate dehydrogenase (LDH) level. These regimens vary in intensity according to risk for relapse and include high‐dose methotrexate for virtually all patients (except LMB Group A) with the methotrexate dose escalated according to risk. Additionally, high‐dose cytarabine is included for the highest‐risk patients; these high‐dose cytotoxic therapies distinguish pediatric regimens from those utilized in adults [4].

Pre‐rituximab event‐free survival (EFS) for Group A/R1 (fully resected disease) was 94%–98%, and for lower‐risk Group B or R2 (unresected limited‐stage or low LDH), EFS of 94%–97% were reported on the BFM and LMB regimens [4, 10, 11, 12]. More recently, patients with higher‐risk Group B/R3 and Group C/R4 disease were included in an international randomized clinical trial that evaluated the efficacy of combining rituximab with a modified LMB96 chemotherapy backbone. This study enrolled patients in North America and across much of Europe, demonstrating superior 3‐year EFS for those patients randomized to receive rituximab (93.9% vs. 82.3% with 95% confidence intervals of 89.1–96.7 vs. 75.7–87.5) [9]. Ultimately, the combination of rituximab with either the LMB or BFM backbone achieves cure for the vast majority of children and adolescents with HGBL, including the highest risk patients. In sharp contrast, patients with relapsed/refractory disease are extremely difficult to salvage and mortality rates remain exceedingly high [13, 14, 15, 16].

Current/future translational and clinical research focuses on identifying distinctions in lymphoma biology that may help leverage potential benefits in novel immunotherapeutic or targeted molecular strategies. Improving salvage rates and potentially identifying subsets of patients in whom de‐escalation of these intensive cytotoxic therapies remain critical gaps to bridge for childhood and adolescent HGBL. Table 2 provides a select list of phase II and III clinical trials for AYA with mature B‐cell lymphomas including both front‐line and salvage regimens. In low‐income countries, particularly in equatorial regions of Africa, curative rates for children and AYA with Burkitt lymphoma remain unjustly low, representing a resounding area of need for the global oncology community [17, 18] Additionally, identifying which young adults may benefit from pediatric‐style regimens potentially represents an opportunity for improving curative outcomes for the AYA population.

**TABLE 2:** Selected ongoing phase 2 and 3 clinical trials for AYA with mature B‐cell NHL.

Study	Region	Age range	Study regimen
NCT03206671 (Phase 3)	Northern Europe *Front line*	Up to 18 years	Risk groups R1 and stage I/II R2: substitution of anthracyclines with rituximab window in patients with limited‐stage disease treated with BFM chemotherapy backbone for. Risk group stage III R2: randomization of rituximab window with backbone chemotherapy for stage III R2 group. Risk groups R3/R4: randomization of one versus seven doses of rituximab plus chemotherapy backbone for patients with advanced‐stage disease (B‐NHL 2013).
NCT05253495 (Phase 2)	United States *Front line*	3–39 years	Addition of polatuzumab vedotin to rituximab + LMB chemotherapy backbone with reduction of anthracycline (RADICAL).
NCT05049473 (Phase 2)	Spain *Front line*	18 and older	Rituximab plus multi‐agent chemotherapy for adults with Burkitt lymphoma/leukemia and high‐grade B‐cell lymphomas (BURKIMAB‐14).
NCT04546620 (Phase 2)	United Kingdom *Front line*	16 and older	Acalabrutinib plus R‐CHOP for patients with DLBCL (REMoDL‐A).
NCT04759586 (Phase 3)	United States *Front line*	2 and older	Randomization of nivolumab with rituximab plus multi‐agent chemotherapy (with or without radiation therapy) for PMBCL (ANHL1931).
NCT05533775 (Phase 1/2)	US, Europe, Asia *Relapsed/refractory*	0.5–30 years	Glofitamab plus ICE chemotherapy for relapsed/refractory mature B‐NHL (iMATRIX‐GLO).
NCT02393157 (Phase 2)	United States *Relapsed/refractory*	3–31 years	Obintuzumab plus ICE chemotherapy for relapsed/refractory mature B‐NHL (O‐ICE).
NCT05255601 (Phase 1/2)	US, Europe, Australia *Relapsed/refractory*	Up to 30 years	Relatlimab plus nivolumab for NHL including DLBCL and PMBCL but excluding aggressive B‐cell lymphomas including Burkitt lymphoma (RELATIVITY‐069).
NCT03210662 (Phase 2)	United States *Relapsed/refractory*	18 and older	Pembrolizumab plus external beam radiation therapy for relapsed/refractory NHL including DLBCL & PMBCL.

Abbreviations: AYA, adolescents and young adults; B‐NHL, B‐cell non‐Hodgkin lymphomas; BFM, Berlin‐Frankfurt‐Münster consortium; DLBCL, diffuse large B‐cell lymphoma, and gray‐zone high‐grade B‐cell lymphomas); LMB, lymphomes malins B (French consortium); PMBCL, primary mediastinal B‐cell lymphoma; US, United States of America.

## 2 DLBCL IN AYA

There are differences between DLBCL in children as compared with adults. DLBCLs account for about 30%–40% of NHL in adults, but only about 15% of NHLs in children [19]. In both pediatrics and adults, there is a slight increase in incidence in males compared with females. The frequency of the activated B‐cell subtype increases with age [20]; in pediatrics, almost all DLBCLs are of germinal center B‐cell origin, whereas the activated B‐cell subtype accounts for about 25% of adult cases. Management of DLBCL is also different between pediatrics and adults. Adult trials generally separate DLBCL from Burkitt lymphoma, whereas pediatric trials include patients with both DLBCL and Burkitt lymphoma and treat them the same. In adults, the combination of rituximab, cyclophosphamide, doxorubicin, vincristine, and prednisone (R‐CHOP) is considered the standard of care for most patients [21, 22]. In contrast, in children, dose‐intense, multi‐agent chemoimmunotherapy regimens, such as those used to manage Burkitt lymphoma, have been the standard [10, 12, 23] In addition, high‐dose systemic methotrexate is routinely administered to children but is used selectively in adults. The prognosis is better in children, with long‐term event‐free survival rates of more than 90%, whereas in adults, it is about 70% and even lower in the elderly.

### 2.1 Treatment of DLBCL in AYA

R‐CHOP remains a standard chemoimmunotherapy regimen for most adults with DLBCL. To improve outcomes, many drugs have been added to R‐CHOP, including lenalidomide [24, 25, 26], bortezomib [27, 28], everolimus [29], ibrutinib [30], obinutuzumab [31], and polatuzumab [32]. Until polatuzumab, all have failed to improve outcomes meaningfully enough to achieve regulatory approval.

The Groupe Etude Lymphoma Francaise (GELA) LNH03‐2B trial may be the only prospective clinical trial performed specifically in the AYA population with DLBCL [33]. The trial compared R‐CHOP with a dose‐intensive, multi‐agent regimen in young adult patient population aged 18–59 years with previously untreated DLBCL and an age‐adjusted international prognostic index of 1. Patients were randomly assigned to receive either either cycles of R‐CHOP or the R‐ACVBP regimen. In the R‐CHOP arm, intrathecal methotrexate was administered during the first four cycles. R‐ACVBP consisted of four cycles of rituximab, doxorubicin, high‐dose cyclophosphamide, vindesine, bleomycin, and prednisone administered in a dose‐intensive fashion every 2 weeks with growth factor support. This was followed by 2 cycles of high‐dose methotrexate 3 grams per square meter IV, then by four cycles of rituximab, ifosfamide, and etoposide, then by two cycles of cytarabine 100 mg per square meter subcutaneously daily for 4 days.

The LNH03‐2B trial demonstrated that, as compared with R‐CHOP, the R‐ACVBP regimen had higher rates of both efficacy and toxicity. One hundred ninety‐six patients were treated on the R‐ACVBP arm and 183 on the R‐CHOP arm. While response rates were similar between the two groups (overall response rate with R‐ACVBP 90% vs. R‐CHOP 87%; complete response rates 83% and 80%, respectively), time‐to‐event endpoints were superior in the R‐ACVBP group (3‐year event‐free survival 81% and 67%, respectively; 3‐year progression‐free survival 87% and 73%, respectively, and 3‐year overall survival 92% and 84%, respectively). Both hematologic and non‐hematologic toxicities were more frequent and more severe in the R‐ACVBP group. Although R‐ACVBP was superior, the higher toxicity rate and lack of availability of vindesine prevented this regimen from ever being utilized in the United States. Nevertheless, these data do suggest that, in younger patients able to tolerate more aggressive treatment, a more intensive multiagent chemoimmunotherapy regimen may lead to better outcomes than R‐CHOP.

**FIGURE 1:**
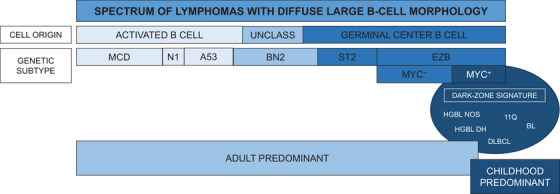
**Spectrum of lymphomas with diffuse large B‐cell morphology in relationship to cell origin and genetic subtype**. Diffuse large B‐cell lymphoma (DLBCL) derives from activated B cells or germinal center B cells, with a small subset of cases remaining unclassified (UNCLASS). Many cases of DLBCL in adults may be classified more precisely according to genetic subtype, with the 7 genetic subtypes depicted above—MCD, N1, A53, BN2, ST2, and EZB. The EZB subtype is further sub‐categorized as *MYC* rearranged (MYC^+^) versus not (MYC^–^). More recent genetic characterization of aggressive DLBCL in adults has shed light on a dark‐zone genetic signature which is shared by double‐hit high‐grade B‐cell lymphoma (HGBL DH) with *MYC* and *BCL2* rearrangements, DLBCL with aggressive clinicopathological features, high‐grade B‐cell lymphoma not otherwise specified (HGBL NOS), Burkitt lymphoma (BL), and presumably HGBL with 11q aberrations (11Q) as well. Defining the biological overlap between childhood and adult DLBCL is a key priority, as it will inform optimal therapeutic strategies for adolescent and young adults with mature B‐cell lymphomas.

The relevance of these findings may have particular impact for adult patients with molecular HGBL gene expression profiles, for whom outcomes with R‐CHOP are significantly worse [34]. Recent data have shed light on a dark‐zone genetic signature in adults that is shared by a subset of patients with aggressive DLBCL of germinal center origin, HGBL with *MYC* and *BCL2* rearrangements, and Burkitt lymphoma [35]. These patients represented less than 15% of an adult cohort of DLBCL and were characterized by significantly worse treatment outcomes. Whether they represent a subset of patients with high‐grade disease analogous to pediatric patients with mature B‐cell NHL is an unanswered question (Figure 1). Biological discovery in this realm may help identify the subset of adults with high‐grade DLBCL/HGBL for whom intensification of chemoimmunotherapy may have the potential to improve curative outcomes as has been achieved in pediatric patients over the past quarter century.

## 3 PRIMARY MEDIASTINAL B CELL LYMPHOMA

Primary mediastinal B‐cell lymphoma (PMBCL) is rare subtype of NHL with an age distribution that is almost exclusively within the AYA range. The rare nature of PMBCL has led to significant gaps in our understanding of the disease biology as well as extremely limited prospective trials to guide clinical management. PMBCL represents a case for “lumping” rather than “splitting” pediatric and adult therapeutic strategies, and collaborations between pediatric and adult oncology teams provide a tremendous opportunity to address these unmet needs.

Although previously considered a subtype of DLBCL, PMBCL is now recognized as a unique clinical and pathologic entity [6]. Treatment for both children and adults with PMCBL includes intensive chemotherapy and, in some cases, radiation with long term PFS ranging from 70%–85% in multicenter studies [36]. There is an urgent need to improve outcomes and develop therapies with a lower risk for long‐term toxicities. One of the molecular hallmarks of PMBCL is copy number alterations in 9p24.1 which result in upregulation of PD‐L1 [37]. PD‐1 inhibitors including pembrolizumab and nivolumab, as well as the anti‐CD30 antibody drug conjugate brentuximab vedotin, have shown efficacy in the relapsed setting however the role for these agents in the upfront setting is not defined [38, 39].

To address this, collaborative pediatric and adult consortia are leading a randomized phase III trial evaluating the addition nivolumab to standard therapy (ANHL1931, NCT04759586). This trial, which includes adult and pediatric patients, was developed as a collaboration between the Children's Oncology Group and the Alliance for Clinical Trials in Oncology with input from all NCI cooperative groups. There are several elements of the design of this trial that were developed to reduce selection bias and maximize enrollment given the rare nature of PMBCL: (1) the trial allows for the treating physician to choose between two chemotherapy backbones (DA‐EPOCH‐R and R‐CHOP). This allows centers to participate regardless of their preferred chemotherapy approach; (2) patients can enroll on study after receiving one cycle of standard therapy off‐study. This permits the inclusion of patients who urgently need to start cycle 1 prior to enrollment; (3) the study is open across the NCI National Clinical Trials Network including NCI Community Oncology Research Program sites; (4) the statistical design is adjusted given the rare nature of the disease with a one‐sided alpha of 0.025. This allows us to conduct the trial with approximately 200 patients. These approaches and the lessons learned from this trial could potentially be applied to future AYA trials in other rare lymphomas. ANHL1931 opened to enrollment in June 2021 and is expected to accrue over 4 years.

There remain several unanswered questions in PMBCL, many of which we are hoping to address within the ANHL1931 trial. While PET/CT is routinely used in PMCBL the prognostic utility of imaging at interim timepoints and at the end of therapy is not defined. We will be collecting imaging on ANHL1931 and utilizing traditional and novel approaches to understand how best to interpret FDG PET in PMBCL. Biomarkers of response are also needed. We will be collecting baseline tumor for molecular profiling and peripheral blood to study circulating tumor DNA. Lastly, we will be adding patient reported outcomes to this trial to better understand how treatment impacts health‐related quality of life. We hope this trial can address many of the key questions in PMBCL and serve as a model for collaboration in AYA oncology.

## 4 NOVEL AGENTS FOR RELAPSED AND REFRACTORY MATURE B CELL LYMPHOMAS

In recent years, several novel agents have demonstrated benefit in patients with relapsed or refractory mature B cell lymphomas, leading to approval by regulatory authorities and widespread use (Table 3). These agents include the CD79b‐targeting antibody‐drug conjugate polatuzumab vedotin [40], the CD19‐targeting monoclonal antibody tafasitamab in combination with lenalidomide [41], the CD19‐targeting antibody‐drug conjugate loncastuximab teserine [42], the inhibitor of exportin‐1 selinexor [43], and the 3 CD19‐targeting chimeric antigen receptor (CAR)‐modified T‐cell therapies axicabtagene ciloleucel [44, 45], lisocabtagene maraleucel [46, 47], and tisagenlecleucel [48, 49]. In addition, bispecific antibodies have demonstrated activity and appear poised for regulatory approval [50, 51, 52, 53]. The Bruton tyrosine kinase inhibitors also have demonstrated activity in patients relapsed disease but are not approved and are undergoing further study [30, 54, 55, 56].

**TABLE 3:** Selected novel agents for AYA with mature B‐ce.

Novel agent	Mechanism of action	Age range	Trial design	Comments
Polatuzumab vedotin	Anti‐CD79b antibody drug conjugate	19–80 (median 65)	R‐CHOP versus Polatuzumab + R‐CHP (phase 3) front‐line regimen for DLBCL (POLARIX).	Polatuzumab arm with improved PFS, DFS, and EFS. No clear benefit of polatuzumab for patients < 60 years or those with GCB‐DLBCL.
Tafasitamab	Anti‐CD19 monoclonal antibody	41–86 years (median 72)	Tafasitamab + lenalidomide (phase 2) for relapsed or refractory DLBCL (L‐MIND).	ORR 58%, CR 40%. No impact of age on outcome.
Loncastuximab teserine	Anti‐CD19 antibody drug conjugate	interquartile range 56–71 years (median 66)	Loncastuximab (phase 2) for relapsed or refractory DLBCL (LOTIS‐2).	ORR 48%, CR 24%. No impact of age on outcome.
Selinexor	Inhibitor of exportin‐1	35–87 years (median 67)	Selinexor (phase 2) for relapsed or refractory DLBCL (SADAL).	ORR 28%, CR 12%. No impact of age on outcome. ORR for double/triple expressor DLBCL 9.7% versus 40.3%.
Axicabtagene ciloleucel (axi‐cel)	CD19 chimeric antigen receptor T‐cell therapy	23–76 years (median 58, ZUMA‐1) and 21–80 years (ZUMA‐7)	ZUMA‐1 (phase 2) for refractory DLBCL, PMBCL, transformed DLBCL. ZUMA‐7 (phase 3) for early relapse or refractory DLBCL, compared axi‐cel to standard of care.	ZUMA‐1: ORR 82%, CR 54%. No impact of age on outcome. ZUMA‐7: ORR 83%, 24‐month EFS 41% in axi‐cel arm, which was superior to standard of care.
Lisocabtagene maraleucel (liso‐cel)	CD19 chimeric antigen receptor T‐cell therapy	18–86 years (median 63, TRANSCEND NHL) and 20–74 years (median 60, TRANSFORM)	TRANSCEND NHL (phase 2) for relapsed or refractory DLBCL, PMBCL, transformed DLBCL. TRANSFORM (phase 3) for early relapse or refractory DLBCL, PMBCL, transformed DLBCL, compared liso‐cel to standard of care.	TRANSCEND NHL: ORR 73%, CR 53%. No impact of age on outcome. Similar ORR in HGBL. TRANSFORM: ORR 86%, CR 66%, 12‐month EFS 44.5% in liso‐cel arm, which was superior to standard of care.
Tisagenluecleucel (tisa‐cel)	CD19 chimeric antigen receptor T‐cell therapy	22–76 years (median 56, JULIET) and 19–79 years (median 59.5, BELINDA)	JULIET (phase 2) for relapsed or refractory DLBCL and transformed DLBCL. BELINDA (phase 3) for early relapse or refractory DLBCL, PMBCL, transformed DLBCL, compared tisa‐cel to standard of care.	JULIET: ORR 52%, CR 40%. Similar ORR in double‐hit DLBCL. BELINDA: ORR 46.3%, CR 28.4%, in tisa‐cel arm, which was NOT superior to standard of care. EFS was shorter in patients with HGBL.
Glofitamab	CD20xCD3 bispecific monoclonal antibody	21–90 years (median 66)	Glofitamab (phase 2) for relapsed or refractory DLBCL, PMBCL, transformed DLBCL.	ORR 52%, CR 39%. There was no CR among 11 patients with HGBL (two partial responses).
Epcoritamab	CD20xCD3 bispecific monoclonal antibody	20–83 years (median 64)	Epcoritamab (phase 1/2) for relapsed or refractory DLBCL, PMBCL, transformed DLBCL.	ORR 63.1%, CR 38.9%, 6‐month PFS 43.9%.
Ibrutinib	Bruton tyrosine kinase inhibitor	19–88 years (median 63, PHOENIX) and 5–19 years (median 15, SPARKLE)	PHOENIX: Randomization of ibrutinib with R‐CHOP front‐line regimen. SPARKLE: Randomization of ibrutinib with RICE/RVICI for relapsed or refractory B‐NHL, including Burkitt lymphoma (patients 1–30 years).	PHOENIX: EFS and OS were improved on ibrutinib arms for patients younger than 60 years with activated B cell‐like subtype of DLBCL. SPARKLE: No benefit in the ibrutinib arm for pediatric/AYA patients.

Abbreviations: AYA, adolescents and young adults; B‐NHL, B‐cell non‐Hodgkin lymphomas; CR, complete response; DFS, disease‐free survival; DLBCL, diffuse large B‐cell lymphoma; EFS, event‐free survival; GCB, germinal center B cell; HGBL, high‐grade B‐cell lymphoma, ORR, overall response rate; PFS, progression‐free survival; PMBCL, primary mediastinal B‐cell lymphoma, transformed DLBCL, transformed from indolent B‐cell lymphoma;

Most of these novel agents have undergone relatively little study in the AYA population. In the POLARIX trial, patients over the age of 18 years with previously untreated DLBCL were randomly assigned to receive R‐CHOP or polatuzumab plus rituximab, cyclophosphamide, doxorubicin, and prednisone (pola‐R‐CHP, in which vincristine was omitted) [32]. As compared with the group treated with R‐CHOP, the group treated with pola‐R‐CHP had improvements in progression‐free survival, disease‐free survival, and event‐free survival. With a median follow‐up of 28.2 months, there was no difference between the groups in overall survival. Toxicities were quite similar between the groups, with a mild increase in febrile neutropenia and anemia with pola‐R‐CHP. While patients as young as 19 years of age were enrolled in both arms, the median age of the patients in both arms was 65–66 years; 271 of 879 patients (31%) were less than or equal to 60 years of age. Although the overall trial results demonstrated a benefit to pola‐R‐CHP over R‐CHOP, in an exploratory subgroup analysis of the PFS endpoint, the benefit of pola‐R‐CHP appeared confined to those over the age of 60 years, suggesting that the AYA subgroup may not have benefitted.

The combination of tafasitamab and lenalidomide was studied in patients with relapsed or refractory DLBCL in the phase 2 L‐MIND trial [41]. The regimen produced an overall response rate of 58%, complete response rate of 40%, median progression‐free survival of 12 months, and median overall survival of 34 months. The trial focused on an elderly population that was not eligible for, or had already received, high‐dose chemotherapy with autologous stem cell transplant. Thus, the median age of the patients was 72 years, with a range of 41 to 86 years. In a subgroup analysis, there was no impact of age on outcome [57].

Loncastuximab teserine demonstrated benefit in patients with relapsed or refractory DLBCL based on the results of the LOTIS‐2 phase 2 trial [42]. The drug achieved an overall response rate of 48%, complete response rate of 24%, median progression‐free survival of 5 months, and median overall survival of 10 months. This trial did not have any AYA patients included, as the median age was 66 years, and the range was 56–71 years. In a subgroup analysis, age had no impact on the results.

Selinexor was studied in DLBCL in the SADAL trial [43]. It achieved an overall response rate of 28%, complete response rate of 12%, median progression‐free survival of 3 months, and median overall survival of 9 months. The median age of the patients was 67 years, with a range of 35–87 years. In subgroup analysis, age had no impact on the results.

In contrast to the above trials of novel agents, which tended to enroll elderly populations, CAR T‐cell trials have included a higher proportion of patients in the AYA range. In both the ZUMA‐1 and ZUMA‐7 trials of axi‐cel, the median age of the patients was 58 years, with a range of 23–76 years in ZUMA‐1 and 21–80 years in ZUMA‐7.[44, 58] In the TRANSCEND NHL 001 trial of liso‐cel in patients with at least 2 prior therapies, the median age was 63 years, with a range of 54–70 years [46]. In the TRANSFORM trial of liso‐cel for patients with only one prior therapy, the median age was 60 years, with a range of 20–74 years [47]. And in the JULIET trial of tisa‐cel, the median age was 56 years, with a range of 22–76 years [49]. Subgroup analyses of the ZUMA‐1 and TRANSCEND NHL 001 trials have looked at outcomes based on age greater or less than 65 years and found no difference; however, analysis specifically of the AYA population has not been performed, to the authors’ knowledge.

Selected trials of bispecific antibodies in patients with relapsed or refractory DLBCL have enrolled patients with median ages in the late 60s, including patients in the AYA range [50, 51, 52, 53]. To the authors’ knowledge, subset analyses of the AYA population have not specifically been performed.

The Bruton tyrosine kinase inhibitor ibrutinib has shown promise in AYA patients with DLBCL. In the PHOENIX trial, patients with previously untreated DLBCL were randomly assigned to receive R‐CHOP with or without ibrutinib [30]. While the overall trial results did not demonstrate a benefit to the addition of ibrutinib, in the subgroup of patients less than 60 years of age, both event‐free and overall survival were superior in patients treated with ibrutinib. This trial formed the rationale for the ongoing ESCALADE trial of acalabrutinib combined with R‐CHOP in treatment‐naïve patients aged 16–75 years with the activated B‐cell subtype of DLBCL [59]. Notably, the SPARKLE trial also failed to demonstrate benefit of adding ibrutinib to RICE/RVICI salvage therapy in children and young adults aged 1–30 years with relapsed or refractory mature B‐cell NHL – possibly reflecting lack of major contribution of BCR signaling to disease in this population [60].

## 5 CONCLUSIONS

Currently, a young adult with mature B cell lymphoma could receive vastly different therapy depending on whether they enter the Emergency Department of a children's hospital versus general oncology service. In some cases (e.g., DLBCL), biology between pediatric and adult lymphoma are distinct. In others (e.g., PMBCL), common origins and biology warrant common approaches. Optimizing therapy for adolescents and young adults remains a major challenge that require creative solutions, including engineering study groups to combine biologically comparable adult and pediatric populations and developing effective salvage strategies that will ultimately be required for investigations of front‐line dose reduction. While collaborative clinical trial efforts have catalyzed improved survival in AYA with mature B cell lymphoma, many challenges opportunities for improving outcomes remain (Table 4).

**TABLE 4:** Priorities to improve outcomes for mature B cell lymphoma in AYA patients.

Refined biological characterization to inform optimal therapy and risk stratification	
Collaboration between pediatric and adult cooperative groups to optimize enrollment of AYA in clinical trials.	
Harmonized evidence‐based treatment guidelines for AYA receiving care at pediatric or adult institutions.	
Coordinated clinical research focus to identify and prioritize effective novel therapeutic salvage strategies.	
Long‐term studies to evaluate quality of life and supportive care needs for patients who survive mature B cell lymphoma as children and young adults.	
Improving outcomes and decreasing disparities in lower and middle‐income settings, where the majority of mature B NHL arises	

## AUTHOR CONTRIBUTIONS

All authors wrote the manuscript and gave final approval.

## CONFLICT OF INTEREST STATEMENT

CEA: Advisory board, Sobi. Research support, Genentech. Other authors have no conflict of interest to disclose.

## FUNDING INFORMATION

The authors received no specific funding for this work.
